# Examining the Effect of *Notocactus ottonis* Cold Vacuum Isolated Plant Cell Extract on Hair Growth in C57BL/6 Mice Using a Combination of Physiological and OMICS Analyses

**DOI:** 10.3390/molecules28041565

**Published:** 2023-02-06

**Authors:** Junko Shibato, Fumiko Takenoya, Ai Kimura, Cheol Woo Min, Michio Yamashita, Ravi Gupta, Sun Tae Kim, Randeep Rakwal, Seiji Shioda

**Affiliations:** 1 Department of Functional Morphology, Shonan University of Medical Sciences, 16-48 Kamishinano, Totsuka-ku, Yokohama, Kanagawa 244-0806, Japan; 2 Department of Sport Sciences, School of Pharmacy and Pharmaceutical Sciences, Hoshi University, 2-4-41 Ebara, Shinagawa-ku, Tokyo 142-8501, Japan; 3 Department of Plant Bioscience, Life and Industry Convergence Research Institute, Pusan National University, Miryang 50463, Republic of Korea; 4 College of General Education, Kookmin University, Seoul 02707, Republic of Korea; 5 Institute of Health and Sport Sciences, University of Tsukuba, 1-1-1 Tennodai, Tsukuba, Ibaraki 305-8574, Japan

**Keywords:** *Notocactus ottonis*, cactus, cold vacuum extraction, hair growth, animal model, gene, protein, metabolite, bioinformatics, health

## Abstract

The biological and psychological importance of hair is recognized worldwide. Molecules that can promote the activation of hair follicle stem cells and the initiation of the growth phase have been subjects of research. Clarifying how hair regeneration is regulated may help to provide hair loss treatments, including cosmetic and even psychological interventions. We examined the hair-growing effects of a cell extract (CE) obtained from cactus *Notocactus ottonis* by the cold vacuum extraction protocol, by investigating its hair-growing effects, relevant mechanisms, and potential factors therein. Using male C57BL/6 mice, vehicle control (VC: propylene glycol: ethanol: water), MXD (minoxidil, positive control), and *N. ottonis* CE (N-CE, experimental) were applied topically to the backs of mice. The results showed that MXD and N-CE were more effective in promoting hair growth than VC. An increase in number of hair follicles was observed with N-CE in hematoxylin-eosin-stained skin tissue. The metabolite composition of N-CE revealed the presence of growth-promoting factors. Using mouse back whole-skin tissue samples, whole-genome DNA microarray (4 × 44 K, Agilent) and proteomics (TMT-based liquid chromatography-tandem mass spectrometry) analyses were carried out, suggesting the molecular factors underlying hair-promoting effects of N-CE. This study raises the possibility of using the newly described *N. ottonis* CE as a hair-growth-promoting agent.

## 1. Introduction

Hair plays an important role in maintaining biological functions such as protection and body temperature maintenance, as well as psychological health. Thus, even if hair loss is not a fatal disease, it is known to negatively impact people psychologically, and as its incidence increases so does the consumer market for hair growth products [1]. Currently, there are only two drugs approved by the U.S. Food and Drug Administration (FDA) for the treatment of hair loss: minoxidil (MXD) and finasteride; the details of MXD’s mechanism of action are unknown, but it is known to prolong the duration of the hair growth phase by inducing vasodilation in the scalp [2,3,4,5]. Finasteride has been demonstrated to prevent androgenic alopecia by inhibiting 5α-reductase activity, which affects the male hormone metabolism [6]. However, side effects have been reported with MXD and finasteride use [7,8]. These issues have led to an increased interest in recent years in adjunctive and alternative therapies using safe and effective natural products.

Our research group has been utilizing a low-temperature vacuum extraction approach for obtaining both a solution and a powder residue of agricultural products (whole plant and plant tissues). The aim of this novel approach is to (1) examine methods for effective utilization of the extracted products and thereby the use of agricultural products or by-products, and (2) further analyze potentially useful molecular components present in these residues. To note, the low-temperature vacuum extraction method differs from the conventional steam distillation method in that it produces almost 100% of the (aroma) oil and cell extract (CE) contained in the raw (plant) material by extraction at a low temperature of 30 to 40 °C under vacuum. CE is expected to be used not only for aroma and beauty, but also for food ingredients and cosmetic materials, since active ingredients such as polyphenols that cannot be extracted by steam distillation, can be detected [9]. 

In the course of our research on the use of this low-temperature vacuum extraction protocol, we found that the cactus *N. ottonis* CE (N-CE) had a hair-growth-promotion effect. Cactus has been used traditionally since ancient times for medicinal and other purposes. Recently, cactus has been attracting attention for its nutritional and medicinal components, as well as functional ingredients for cosmetic purposes, and is expected to be used as a raw material in the food, cosmetic, and pharmaceutical industries [10,11,12,13]. However, there are a few research reports investigating the effects of cactus on hair growth. 

This study attempted to provide new basic data on hair growth effects and mechanisms therein for the use of N-CE as a new hair-growth-promoting agent by conducting comparative experiments with treatment by MXD, approved by the US FDA. The gene and protein expression data are publically available at NCBI GSE222210 and ProteomeXchange identifier PXD039218.

## 2. Results

### 2.1. Hair Growth Promoting Action

**Figure 1:**
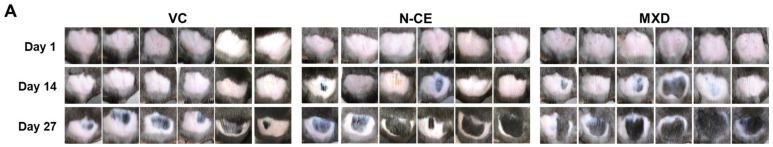

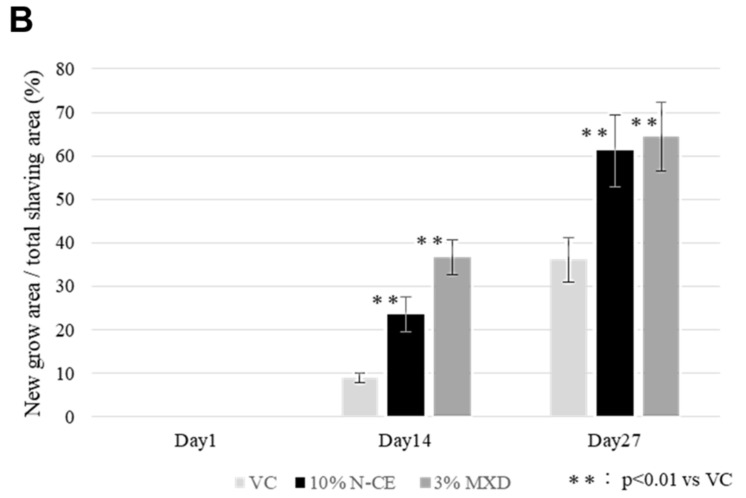
(**A**) The promotion of hair growth in C57BL/6N mice. VC (propylene glycol: ethanol: water (5:3:2)) and N-CE, MXD were applied to the back skin of C57BL/6N male mice for 27 days and the effect on hair growth was observed. (**B**) The area of newly grown hairs was measured by ImageJ. The x-axis represents time and the y-axis represents the area of newly grown hairs as a percentage of the total area. Results were presented as mean ± SD ** *p* < 0.01 when compared to the respective VC values by Student’s *t*-test (n = 6).

Figure 1 shows the percentage of hair-growing areas in mice 14 and 27 days after application of the adjusted sample (N-CE, MXD and a vehicle control-VC) in the mouse back skin hair-growing experiment (Figure 1A). Fourteen days later, the hair-growing rate of N-CE was slightly lower than that of MXD, but 27 days later, the hair-growing effect was as high as that of MXD (Figure 1B).

### 2.2. Optical Microscopic Observation with H&E Staining

**Figure 2:**
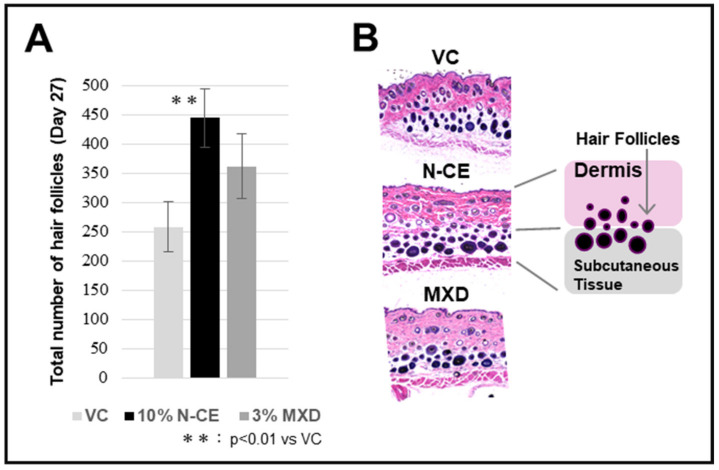
The effect of N-CE and MXD treatments on the number of hair follicles in the C57BL/6 mice. Hematoxylin- and eosin-stained photomicrographs of different groups of whole-layer skin tissue sections of the mouse back. (**A**) The total number of follicles in the dermis and subcutaneous tissue and the number of follicles in the subcutaneous tissue only was measured in section photographs, and the average number of follicles was calculated and plotted. (**B**) Micrographs of the hair follicles of VC control, N-CE, and MXD group. Results were presented as mean ± SD ** *p* < 0.01 when compared to the respective VC values by Student’s *t*-test (n = 6).

The data shown in Figure 2A were obtained 27 days after the start of the experiment, and the graph shows the follicle average (n = 6). Follicle number increased in both N-CE and MXD compared with VC, but a significant (*p* < 0.01) increase was observed in N-CE. The observation of the number of hair follicles in the back skin of mice evaluated for hair growth using an optical microscope is shown in Figure 2B. 

### 2.3. Results of the Component Analysis

CE-TOFMS analysis of N-CE was performed for substances registered in the HMT Metabolite Library and the Known–Unknown Peak Library. As a result, 89 peaks (62 cations and 27 anions) were detected. Quantitative values were calculated for 10 (8 cations, 2 anions) of the detected peaks among the target component metabolites (Table 1). The substances listed in Table 1 were examined and several of them were found to be involved in hair growth. Substances involved in hair growth are highlighted in gray, and substances contained in hair care products such as shampoos and conditioners are shaded. Adenosine was detected as a substance related to hair growth. Adenosine has been commercialized as “Medicated Adenogen EX” (Shiseido) [14]. In addition, pantothenic acid, pyridoxal, pyridoxamine, pyridoxine, riboflavin, and other members of the vitamin B family were also abundant [15]. Pantothenic acid, in particular, is also found in many shampoos and hair growth products [16]. These results clearly indicate that N-CE contains numerous substances involved in hair growth and conducive towards hair care.

### 2.4. Results of the DNA Microarray Analysis

**Figure 3:**
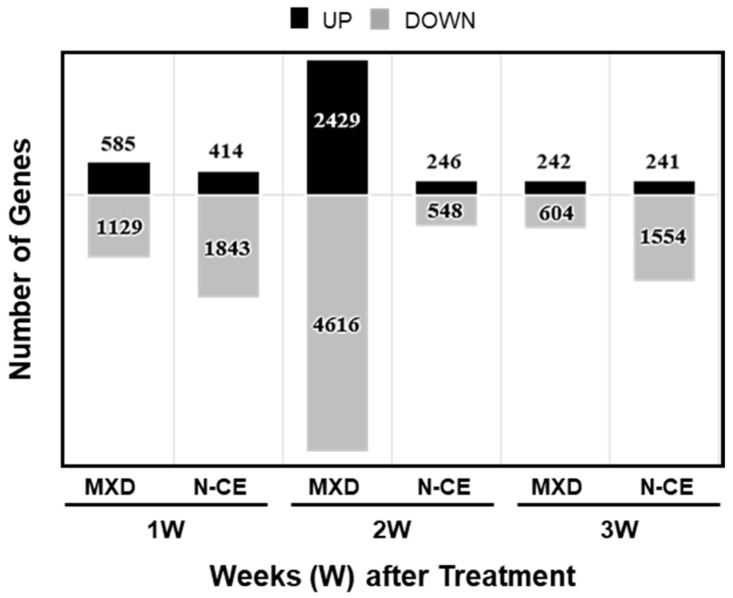
Time course profiling of the genes up- and down-regulated in the N-CE and MXD group 1 week, 2 weeks, and 3 weeks after treatment (see also Table 2 and Table 3 below). DNA microarray was performed as described in the Section 4.

The whole-genome DNA microarray data of treated (MXD and N-CE, over VC) back whole-skin tissue of C57BL/6J mice were submitted to NCBI’s GeneExpression Omnibus under the GEO series accession number GSE222210 (https://www.ncbi.nlm.nih.gov/geo/query/acc.cgi?acc=GSE222210, accessed on 29 January 2023). Figure 3 shows the number of genes up-regulated more than 1.5-fold or down-regulated less than 0.75-fold at each time point (week after treatment: 1W, 2W, and 3W; the DNA microarray sample dye-swap chip combinations are indicated). Overall, more genes were down-regulated than up-regulated, but the number of variable genes was higher after 2 weeks of MXD treatment. With MXD there was a peak in gene expression at 2 W with 2429 and 4616 up- and down-regulated genes, respectively, followed by a much lesser change in gene expression at 3W. In the case of N-CE, a higher number of gene expressions at 1W were almost halved at 2W followed by a large increase in number of only down-regulated genes at 3W. Table 2 and Table 3 lists the top 20 variable genes changed by MXD and N-CE at 1W, 2W, and 3W post-treatment.

MXD changed gene expressions—Most of the up-regulated gene groups fell into the cytokine, keratin, cell-adhesion, and cell-proliferation categories. In relation to hair growth, increased expression of *Lef1* (a papilla cell marker [17]) and *Dsg4* (an important mediator of follicular keratinocyte cell adhesion [18]) were observed. In the decreased gene expression group, many genes were classified under muscle contraction, iron homeostasis, and cell proliferation, and others were related to auto phagosomes and coagulation (Figure 3).

N-CE changed gene expressions—The categories of genes with increased expression were angiogenesis, autophagy, growth/differentiation, muscle contraction, and cell adhesion, while *FGF2* (hair follicle growth and development [19]) and *PAX6* (melanin formation [20]) were identified as hair-growth-related genes. Cytokines, melanocytes, coagulation, and iron homeostasis were the most frequently down-regulated genes (Figure 3).

### 2.5. Proteome Analysis Results

#### TMT-Based Quantitative Proteomic Analysis

**Figure 4:**
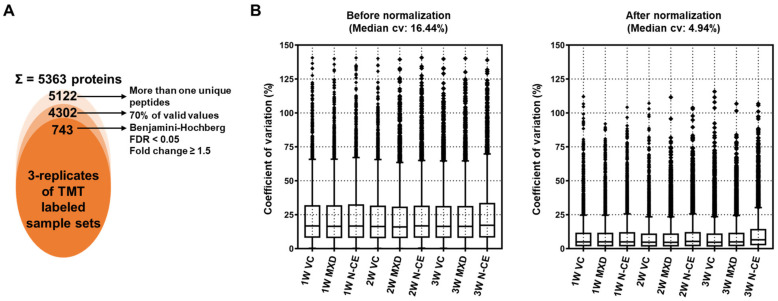
Label-free quantitative proteomic analysis of total proteins extracted from MXD and N-CE groups. (**A**) Venn diagram showing the distribution of the total identified and significantly modulated proteins followed by narrow-down approaches. (**B**) Box plots showing the improvement in the reproducibility of all proteins normalized with pooling intensities. The median CV values of the replicates decreased by 11.5% following normalization by the IRS method.

The mass spectrometry proteomics data were deposited to the ProteomeXchange Consortium via the PRIDE [21,22] partner repository with the dataset identifier PXD039218. For the in-depth proteome analysis, TMT-labeled peptides were further fractionated into 12 fractions by basic pH reversed phase using in-house developed stage-tips. The sequential LC-MS/MS analysis led to the identification of 53,065 peptides and 38,181 unique peptides matched with 5363 protein groups with an average sequence coverage of 24.5% (Figure 4A and Supplementary Table S1). 

**Figure 5:**
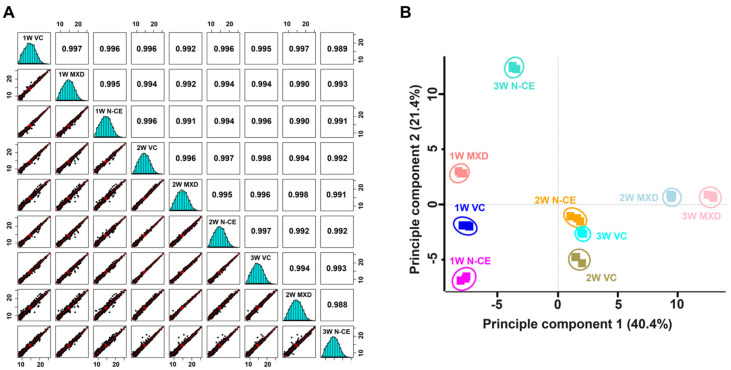
(**A**) Multi-scatter plot indicating the correlation between protein intensities among MXD and N-CE groups sample sets. Reproducibility across the replicate of three samples was revealed by Pearson’s correlation value. (**B**) Principle component analysis of significantly modulated proteins was identified by TMT-based quantitative proteome analysis.

A further removal of potential contaminants and missing values (three values in the three replicates, at least one group) were applied that narrowed down the identification of list of 4302 proteins (Figure 4A). Normalization of protein intensities by internal reference scaling (IRS) method improved the coefficient of variation (CV) values from 16.44% to 4.94% (Figure 4B and Supplementary Table S1). To examine the correlation and variations between two sample sets and the reproducibility of different replicates of the same sample, multi-scatter plot and principal component (PCA) analyses were performed using Perseus software. Moreover, the Pearson’s correlation coefficient values of different samples were in the range of 0.988 to 0.997, further indicating a high degree of correlation among different sample sets (Figure 5A). 

PCA plot analysis showed that the clear separation of samples in principle component 1 that accounted for 40.4% for the total variation (Figure 5B). Sequential application of Student’s *t*-test controlled by a Benjamini–Hochberg FDR threshold of 0.05 led to the identification of 743 (with >1.5 fold change differences) significantly modulated proteins (Figure 4A and Supplementary Table S1).

MXD changed proteins—In the first week after application, increased protein expression of cytokines, neutrophils, keratin, and blood coagulation/fibrin clot formation were observed, but in the second and third weeks, the majority was keratin-related (Table 4). In terms of hair-growth-related proteins, increased expression was observed for Tchhl1 (keratinocyte proliferation and hair formation [23,24]), Tchh (hair stem formation [25]), Padi3, Padi1 (hair stem differentiation through citrullination of TCHH via PADI1/3 [26]), and Padi4 (regulation of hair stem cell regeneration and proliferation [26]). Most of the down-regulated proteins were related to muscle contraction. 

N-CE changed proteins—The categories of proteins with increased expression were endocytosis, autophagy, and muscle contraction, while Ldb1 (maintenance of hair follicle stem cells [27]), Kctd1 (skin and hair development [28]), and Myo5a (involved in hair color [29,30]) were identified as hair growth related (Table 4). Categories of decreased expression proteins included keratin, iron binding, and actin binding.

### 2.6. Biological Functional Enrichment Analysis

To gain insight into the hair-growth-promoting effects of N-CE, the DNA microarray-based approach was used to analyze a set of genes whose expression was altered by N-CE over time using the DAVID tool for functional categories (KEYWORDS) and pathway (KEGG pathway) analysis using the DAVID tool to profile mRNA expression in the back skin of mice post-treatment. The results of the biofunctional analysis (Functional Category Term) obtained by the DAVID tool were significant (*p*-value ≤ 0.05), and those terms that had a large number of genes classified as “Signal” or “Activator”, for example, were omitted (Table 5 and Table 6). The top gene in the MXD UP (MXD UP: Microarray) category was keratin, which is involved in keratinization, melanin biosynthesis, and other processes related to hair, keratinization, melanin biosynthesis, etc. (Table 5). The top down-regulated genes (MXD Down: Microarray) were those involved in muscle contraction, such as muscle proteins, myosin, and sarcoplasmic reticulum (Table 5). This result was almost similar for the identified protein categories (Table 5). 

On the other hand, the up-regulated genes by the N-CE (N-CE UP: Microarray) category included immunity, cytokines, inflammation, Golgi, citrullination, and homeoboxes (Table 6), while the down-regulated gene (N-CE Down: Microarray) category included keratin, melanin biosynthesis, and muscle proteins, synthesis, and muscle proteins (Table 6). In the case of proteins, keratin and muscle proteins were abundantly included in both the up- and down-regulated categories (Table 6).

## 3. Discussion

The aims of this mouse study were to first identify the hair-growing effects of the cactus (*N. ottonis*) whole above-ground part cell extract (N-CE) isolated using cold vacuum extraction by biophysiochemical methods, and, second, to create a database of underlying metabolites (components), genes, and proteins using omics-based analyses. These data are open and publically available to all researchers wishing to conduct further detailed studies on the novel gene and protein expressions identified therein (GSE222210 and PXD039218). Our obtained data provided support to the efficacy and potential use of a new plant (common cactus)-based treatment for hair growth. Moreover, it also attempted to determine what molecular components may be behind the positive hair-promoting effects of N-CE linking component metabolites contained in N-CE (Table 1) to the whole-genome and -proteome of N-CE compared to the over-the-counter medication MXD (Table 2, Table 3, Table 4, Table 5 and Table 6). In MXD, as expected, the expression of keratin, melanin, and other hair-related genes and proteins increased, while the expression of those related to muscle contraction decreased. This is thought to be due to the fact that MXD’s hair-growth action mechanism of vascular smooth muscle relaxation (vasodilation) leads to an improvement in the blood flow whereby the hair-growth promotion actually occurred. However, with the N-CE we could observe multiple categories of genes and proteins different from those changed under MXD application. The fact that N-CE contained many substances involved in hair growth as a result of component (substance) analysis (Table 1) also suggested the existence of a hair-growth mechanism of action different from that of the MXD.

When examined in terms of ingredients with hair-growth effects, the involvement of adenosine was considered. Adenosine is believed to inhibit platelet aggregation by increasing the concentration of cyclic AMP in platelets via the adenosine A2A receptor, relax vascular smooth muscle, promote angiogenesis and growth factors, and is marketed by Shiseido as the hair-growth agent “Adenogen” [14]. The results of this study showed that N-CE decreased coagulation, increased angiogenesis and growth factors, but did not show a clear decrease in muscle contraction like that observed with MXD. It was suggested that this result may be due to the fact that adenosine has vasodilating but also vasoconstrictive effects [31]. The next possible explanation was the involvement of autophagy. Recently, there have been many reports of hair growth promotion by autophagy activation. It is believed that induction of autophagy induces the hair follicle cycle to enter the growth phase and prolongs the growth phase. Furthermore, it has been shown that the antimalarial agent chloroquine, an autophagy inhibitor, may induce changes in hair color and hair loss [32]. It is suggested that N-CE may have an effect on autophagy-induced hair growth.

## 4. Materials and Methods

### 4.1. Plant Material

The common cactus *N. ottonis* (called ‘Seiōmaru’ in Japanese) was provided by the Gifu Agricultural Technology Center (GATC, https://www.g-agri.rd.pref.gifu.lg.jp/, accessed on 29 January 2023) in Japan. Although the ‘Uchiwa’ cactus (*Opuntia*, commonly called prickly pear of the Cactaceae family) is the best growing cactus in terms of variety, a test for the biological antioxidant potential (BAP; Wismer Co., Tokyo, Japan) revealed higher antioxidant power for *N. ottonis*. This was the main reason to select the ‘Seiōmaru’ cactus (*N. ottonis*) in the present study.

### 4.2. Cell Extract by Low-Temperature Vacuum Extraction Method

Cell extract (CE) was obtained from whole above-ground parts of *N. ottonis* (N-CE) through the low-temperature vacuum extraction method [9]. Briefly, the *N. ottonis* tissues were prepared for extraction by placing them into a container of a low temperature vacuum extraction device (FED-50, F · E · C Ltd., Hyogo, Japan; Yoshimi Medical Farm Co., Ltd., Tokyo, Japan). Stirring was carried out at a temperature of 40 °C for 5 h under vacuum (−90 kPa or less). The vacuum condition was created by an in-house developed water ejector creating a vacuum state by the high-speed water flow, resulting in the evaporation. The evaporating gas in the apparatus container was cooled via a condenser and recovered as a liquid (N-CE). To note, this principle is highly efficient in separating and recovering both the liquid (cell extract) and the solid (cell powder). The obtained N-CE was used in the following experiments as explained below. 

### 4.3. Animal Experiment for the Evaluation of Hair Growth Using Mouse Back Skin Model

The back skin model of C57BL/6J mice was used to evaluate the hair growth-promoting effects of N-CE. Briefly, the experimental protocols were reviewed and approved by the Animal Care Committee of Hoshi University School of Pharmacy and Pharmaceutical Sciences (19-089). Male C57BL/6 mice (8 weeks-old) were purchased from Tokyo Laboratory Animals Science Co., Ltd. (Tokyo, Japan). The animals were housed at the Animal Institution in Hoshi University. They were maintained in cages in a ventilated animal room with controlled temperature and relative humidity with a 12 h light: 12 h dark cycle with free access to water and rodent chow. MXD has been used to treat alopecia [5] and is commonly used as a positive control in many hair studies, so it was used as a comparison in our study. To increase the penetration of the test sample, propylene glycol:ethanol:water (5:3:2) was used as the vehicle control group (VC), and preliminary experiments were conducted with N-CE at 1, 10, and 20% concentrations. C57BL/6 mice were divided into 3 groups (6 mice per group): N-CE: 10%, MXD: 3%, and VC. Briefly, starting the day after shaving, 200 μL of the adjusted sample was applied daily to the backs of the mice for observation and photography. Photographs of mouse backs were analyzed using ImageJ software. The percentage of hair-growing area to shaved area of mice at different stages was calculated using (hairy skin area/total shaved skin area) × 100. Data were expressed as mean ± standard deviation (SD) (n = 6).

### 4.4. Histological Observations

The C57BL/6J mice were deeply anesthetized with three types of mixed anesthetic agents (5 mL kg^−1^, i.p.) and perfused transcardially with phosphate-buffered saline (PBS) followed by 4% paraformaldehyde (PFA, Merck) in 0.1 M phosphate buffer (PB). Post-dissection, dorsal whole skin tissues of were fixed in 4% PFA solution for 3 days and replaced with PBS. Paraffin sections were dehydrated for embedding in paraffin (1:1 mixture of Paraffin Wax II 60: Sakura Finetek, Tokyo, Japan, and Pathoprep568: Wako, Tokyo, Japan) and permeabilized with xylene. Five μm paraffin sections were stained with hematoxylin/eosin (Mayer’s Hematoxylin Solution (Fujifilm, Wako, Japan) for 5 min, 1% Eosin Y Solution (Fujifilm, Wako, Japan), and after 1 min of treatment, the samples were dehydrated and sealed with a cover glass. Images were captured by a BZ-X 710 brightfield system (Keyence) at ×20 magnification. Photomicrographs of whole mouse back skin tissue sections were analyzed using ImageJ software. Total number of follicles in the dermis and subcutaneous tissue and the number of follicles in the subcutaneous tissue only were measured in the section photographs at different stages, and the mean number of follicles was calculated and graphed. Data were expressed as mean ± standard deviation (SD) (n = 6).

### 4.5. Component Metabolite Analysis

N-CE was concentrated 400-fold in an evaporator, and 20 μL of aqueous solution prepared to a concentration of 1000 μM of internal standard for 80 μL sample was added, stirred, and transferred to an ultrafiltration tube (Ultrafree MC PLHCC, HMT, centrifugal filter unit 5 kDa). This was centrifuged (9100× *g*, 4 °C, 60 min) and subjected to ultrafiltration. Samples were subjected to CE-TOFMS (capillary electrophoresis-time-of-flight mass spectrometry at Human Metabolome Technologies (HMT; Tsuruoka, Japan): 7100 CE System, 6210 TOF-MS, Agilent Technologies Inc., Santa Clara, CA, USA) (Capillary: fused silica capillary i.d. 50 μm × 80 cm). Peaks detected by CE-TOFMS were automatically extracted using the automatic integration software MasterHands ver. 2.17.1.11 (developed by Keio University, Japan, [33]) for peaks with a signal-to-noise (S/N) ratio of 3 or higher, and mass-to-charge ratio (*m*/*z*), peak area value, and migration time (MT) were obtained. The obtained peak area values were converted to relative area values using the formula relative area value = area value of peak of interest/area value of internal standard × sample. The detected peaks were searched against all substances registered in the HMT Metabolite Library and the Known–Unknown Library based on the *m*/*z* and MT values. Tolerance for the search was set to ±0.5 min for MT and ±10 ppm for *m*/*z*. Quantitative values were calculated for 10 (8 cation and 2 anion) detected peaks among the target metabolites. The calibration curves were based on peak areas corrected by internal standards, and concentrations were calculated for each substance as a single-point calibration of 100 µM (200 µM internal standard; HMT).

### 4.6. DNA Microarray Analysis

For DNA microarray analysis sampling, spinal dislocation was employed and the skin samples were collected. The back whole skin tissue of C57BL/6J mice was ground into a fine powder in liquid nitrogen and stored at −80 °C until RNA isolation [34,35,36]. Total RNA was extracted from powdered mouse skin tissue using the RNeasy Mini Kit (74104, Qiagen, Germantown, MD, USA). To verify the quality of this RNA, yield and purity were determined spectrophotometrically with DS-11 (DeNovix, Wilmington, DE, USA) and confirmed using formaldehyde–agarose gel electrophoresis. To check the quality of the synthesized cDNA using the Affinity Script QPCR cDNA synthesis kit (600559, Agilent Technologies Inc., Santa Clara, CA, USA), PCR reaction was performed to confirm the expression of house-keeping genes (beta-actin or GAPDH) using Emerald Amp PCR Master (RR300A, Takara, Japan). PCR products were separated on a 1.6% agarose gel and visualized with ethidium bromide staining under UV light. Total RNA extracted from mouse skin tissue for each control and treatment (n = 6) was pooled in each group, prior to DNA microarray analysis (Whole Mouse Genome DNA Microarray 4 × 44K, G4122F; Agilent Technologies Inc., Santa Clara, CA, USA). Microarray experiment was as described previously [34,35]. Total RNA (400 ng) was labeled with either Cy3 or Cy5 dye using an Agilent Low RNA Input Fluorescent Linear Amplification Kit (Agilent). Fluorescently labeled targets of control as well as treated samples were hybridized to the same microarray slide with 60 mer probes. A flip labeling (dye-swap or reverse labeling with Cy3 and Cy5 dyes) procedure was followed to nullify the dye bias associated with unequal incorporation of the two Cy dyes into cDNA. Briefly, to select differentially expressed genes by the dye-swap approach, hybridization and wash processes were performed according to the manufacturer’s instructions, and hybridized microarrays were scanned using an Agilent Microarray scanner G2505C (Agilent Technologies Inc., Santa Clara, CA, USA). 

For the detection of significantly differentially expressed genes between control and treated samples, each slide image was processed by Agilent Feature Extraction software (version 9.5.3.1). This program measures Cy3 and Cy5 signal intensities of whole probes. Dye-bias tends to be signal intensity dependent; therefore, the software selected probes using a set by rank consistency filter for dye-normalization. Said normalization was performed by LOWESS (locally weighted linear regression) which calculates the log ratio of dye-normalized Cy3- and Cy5-signals, as well as the final error of log ratio. The significance (P) value based on the propagate error and universal error models. In this analysis, the threshold of significant differentially expressed genes was <0.01 (for the confidence that the feature was not differentially expressed). In addition, erroneous data generated due to artifacts were eliminated before data analysis using the software. 

The functional categories (KEYWORDS) and pathway (KEGG pathway) of the list of variable genes selected by the microarray analysis were analyzed using the Database for Annotation, Visualization and Integrated Discovery (DAVID) v6.8. 

### 4.7. Proteome Analysis

For proteomics the skins were sampled as in Section 4.6 and the finely ground powder in liquid nitrogen that was stored at −80 °C was used for extraction of total proteins exactly as described previously [36]. Briefly, the LB-TT solution [7 M (*w*/*v*) urea, 42 g; 2 M (*w*/*v*) thiourea, 15.2 g; 4% (*w*/*v*), CHAPS, 4.0 g; 18 mM (*w*/*v*) Tris-HCl (pH 8.0), 1.8 mL; 14 mM (*w*/*v*) Trizma base, 169.5 mg, 0.2% (*v*/*v*) Triton X-100, 0.2 mL 50 mM (*w*/*v*) DTT, 771.5 mg, 1% (*v*/*v*) pH 3-10 Ampholyte, 1 mL, and two EDTA-free proteinase inhibitor tablets in a total volume of 100 mL] was used to extract total proteins [36]. Protein concentration was determined with a Pierce™ 660 nm Protein Assay Reagent (Thermo Fisher Scientific, Waltham, Waltham, MA, USA) using bovine serum albumin (BSA) as a standard and a DeNovix DS-11 spectrophotometer (DeNovix, Wilmington, DE, USA). The total proteins solubilized (and stored) in LB-TT were processed for downstream liquid chromatography–tandem mass spectrometry (LC-MS/MS) analysis as described below.

#### 4.7.1. Protein Digestion by Filter-Aided Sample Preparation (FASP)

Protein digestion was carried out using a filter-aided sample preparation (FASP) approach as described in previous studies [37,38]. Briefly, acetone-precipitated proteins (300 μg) were dissolved in 30 μL of denaturation buffer (4% sodium dodecyl sulfate (SDS) and 100 mM dithiothreitol (DTT) in 0.1 M tetraethylammonium tetrahydroborate (TEAB), pH 8.5). After sonication of the sample for 3 min and heating at 99 °C for 30 min, denatured proteins were loaded onto a 30 kDa spin filter (Merck Millipore, Darmstadt, Germany) and diluted with UA buffer (8 M urea in 0.1 M TEAB, pH 8.5) to a final volume of 300 μL. The buffer was washed and exchanged three times using 300 μL of UA buffer by centrifugation at 14,000× *g* for removal of SDS. After removing SDS from the samples, cysteine alkylation was accomplished through the addition of 200 μL of alkylation buffer (50 mM iodoacetamide (IAA), 8 M urea in 0.1 M TEAB, pH 8.5) for 1 h at room temperature in the dark. Then, the buffer was exchanged with UA buffer to TEAB buffer (50 mM TEAB, pH 8.5) in a spin filter unit. The protein was digested with trypsin (enzyme-to-substrate ratio (*w*/*w*) of 1:100) dissolved in 50 mM TEAB buffer containing 5% acetonitrile (ACN) at 37 °C overnight. After overnight digestion, the digested peptides were collected by centrifugation, and the filter device was rinsed with 50 mM TEAB and 50 mM NaCl. The peptide concentrations were measured using the Pierce Quantitative Fluorometric Peptide Assay (Thermo Fisher Scientific, Waltham, MA, USA) according to the manufacturer’s instructions. Moreover, we performed further sample preparation including peptide labeling and fractionation for MS analysis.

#### 4.7.2. Peptide Labeling with Tandem Mass Tags (TMT), Desalting, and Basic pH Reversed-Phase (BPRP) Peptide Fractionation Using Stage-Tip

TMT labeling of digest peptides was performed as described previously [37,38,39] and following the manufacturer’s instructions using a TMT-10plex kit (Thermo Fisher Scientific, Waltham, MA, USA). Briefly, each TMT reagent (0.8 mg) was dissolved in 120 μL of anhydrous ACN, of which 25 μL was added to each channel of samples. Prior to incubation, to each sample, ACN was added to reach 30% of the final concentration, i.e., optimal conditions of TMT labeling it require at least 30% of ACN concentration in each tube (sample or channel). After incubation at room temperature for 1 h, the reaction was quenched with hydroxylamine to a final concentration of 0.3% (*v*/*v*). Finally, samples were combined at equal amounts across all samples, lyophilized to near dryness, and subjected to the desalting procedure. The pooled TMT-labeled peptides were desalted using the HLB OASIS column (Waters, Milford, MA, USA) according to the manufacturer’s instructions. Consequently, dried peptides were reconstituted in 200 μL of loading solution (15 mM Ammonium formate, 2% ACN) and loaded onto stage-tip prepared by packing C18 Empore disk membranes (3M, Bracknell, UK) at the bottom and POROS 20 R2 reversed-phase resin (Thermo Fisher Scientific, Waltham, MA, USA) into a 200 μL yellow tip. Prior to loading the peptides, the stage-tip was washed with 100% methanol and 100% ACN and equilibrated with loading solution. The peptides were loaded and 12 fractions were subsequently eluted with pH 10 buffer solution containing 5, 8, 11, 14, 17, 20, 23, 26, 29, 32, 35, 41, 44, 60, 80, and 100% ACN as described previously. Finally, the 12 fractions were lyophilized in a vacuum centrifuge and stored at −80 ℃ until further LC-MS/MS analysis.

#### 4.7.3. Q-Exactive MS Analysis

Obtained peptides were dissolved in solvent-A (water/ACN, 98:2 *v*/*v*; 0.1% formic acid) and separated by reversed-phase chromatography using a UHPLC Dionex UltiMate^®^ 3000 (Thermo Fisher Scientific, Waltham, MA, USA) instrument [40]. For trapping the sample, the UHPLC was equipped with Acclaim PepMap 100 trap column (100 μm × 2 cm, nanoViper C18, 5 μm, 100 Å) (Thermo Fisher Scientific, Waltham, MA, USA) and subsequently washed with 98% solvent A for 6 min at a flow rate of 6 μL/min. The sample was continuously separated on an Acclaim PepMap 100 capillary column (75 μm × 15 cm, nanoViper C18, 3 μm, 100 Å) at a flow rate of 400 nL/min. The LC analytical gradient was run at 2% to 35% solvent B (100% ACN and 0.1% formic acid) over 90 min, then 35% to 95% over 10 min, followed by 90% solvent B for 5 min, and finally 5% solvent B for 15 min. LC-MS/MS was coupled with an electrospray ionization source to the quadrupole-based mass spectrometer QExactive™ Orbitrap High-Resolution Mass Spectrometer (Thermo Fisher Scientific, Waltham, MA, USA). Resulting peptides were electro-sprayed through a coated silica emitted tip (Scientific Instrument Service, NJ, Amwell Township, USA) at an ion spray voltage of 2000 eV. The MS spectra were acquired at a resolution of 70,000 (200 *m*/*z*) in a mass range of 350–1650 *m*/*z*. The automatic gain control (AGC) target value was 3 × 10^6^ and the isolation window for MS/MS was 1.2 *m*/*z*. Eluted samples were used for MS/MS events (resolution of 35,000) and measured in a data-dependent mode for the 15 most abundant peaks (Top15 method) in the high-mass-accuracy Orbitrap after ion activation/dissociation with Higher Energy C-trap Dissociation (HCD) at 32 collision energy in a 100–1650 *m*/*z* mass range [40]. The AGC target value for MS/MS was 2 × 10^5^. The maximum ion injection time for the survey scan and MS/MS scan was 30 ms and 120 ms, respectively.

#### 4.7.4. Data Processing Using MaxQuant Software and Data Analysis Using Perseus and R Program

The MaxQuant software (version 1.6.17.0, Max Planck Institute of Biochemistry, Munich, Germany) was used to apply for a database search as described previously [38,41,42]. All the three technical replicates of matured and seed-filling stage samples were cross-referenced against the Rat (*Rattus norvegicus*) and Mouse (*Mus musculus*) database (36,180 and 86,074 entries, respectively, https://www.uniprot.org/, accessed on 29 January 2023). TMT data processing of two different data was performed using default precursor mass tolerances set by the Andromeda search engine, which was set to 20 ppm for the first search and 4.5 ppm for the main search. Reporter mass tolerance was set to the minimum of 0.003 Da and the minimum reporter precursor ion fraction (PIF) was set as 0.5. The product mass tolerance was set to 0.5 Da and a maximum of two missed tryptic cleavages were allowed. Carbamidomethylation of cysteine residues and acetylation of lysine residues and oxidation of methionine residues were specified as fixed and variable modifications, respectively. A reverse-nonsense version of the original database was generated and used to determine the FDR which was set to 1% for peptide identifications. Statistical analysis was carried out using Perseus software (ver. 1.6.17.0) (Max Planck Institute of Biochemistry, Munich, Germany) [43]. The normalization of reporter ion intensities followed by the internal reference scaling (IRS) methods was performed as described previously [37,44]. Briefly, we employed the multiple normalizations through Bioconductor of R program in which the first sample loading (SL) normalization among the samples was conducted for corrections of sample loading errors using calculating by mean from the sum value of each TMT channel/sample and the second IRS method was applied among the technical replicates represented by separate MS runs. Missing value imputation was carried out from a normal distribution (width: 0.3, downshift: 1.8) using Perseus software [43,44]. Multiple-Sample test controlled by the Benjamini–Hochberg FDR threshold of 0.05 was applied to identify the significant differences in the protein abundance (≥1.5-fold change).

## 5. Conclusions

The hair-growth-promoting effects of a common cactus cell extract (N-CE) were unraveled by a combination of the cold vacuum extraction protocol and omics tools. Firstly, new information on the component metabolites in the extract of *N. ottonis (N-CE*) was clarified, which, other than known substances known to promote hair growth such as adenosine and vitamin B family members (e.g., pantothenic acid), also identified gibberellic acid (GA), an essential phytohormone implicated in the regulation of several developmental processes. These components need to be studied further alone (i.e., GA) and/or in combination with each other to identify their effects on hair growth. Although some of these components are available as commercial products, evidence-based scientific studies are needed for validation. Secondly, using N-CE on a mouse skin model in conjunction with high-throughput omics techniques, genomics, and proteomics, a database of the molecular changes (genes: *FGF2*, *PAX6*; and proteins: Ldb1, Kctd1, Myo5a) was created. This is a great resource for investigating the multitude of molecules with changed expression is the skin, and with a possibility of discovering pathways and mechanisms underlying such an effect (hair growth promotion). Human studies (using skin) would be an obvious next step but will require knowledge of the key components to be examined, including safety and risks.

## Figures and Tables

**Table 1:** Component metabolites with narrowed down candidates in N-CE.

ID	HMT DB	Relative Area		ID	HMT DB	Relative Area	
	Compound Name	Cell-Extract	Concentration (μM)		Compound Name	Cell-Extract	Concentration (μM)
		N-CE				N-CE	
C_0050	1-Methyl-4-imidazoleacetic acid	3.90 × 10^−5^		C_0087	Kynurenine	1.50 × 10^−5^	
C_0096	2’-Deoxyadenosine 5’-Deoxyadenosine	1.42 × 10^−4^		C_0034	Melamine	7.97 × 10^−5^	
C_0091	2’-Deoxycytidine	6.73 × 10^−4^		A_0072	Mevalonic acid	1.54 × 10^−5^	
C_0099	2’-Deoxyguanosine	1.23 × 10^−4^		C_0089	*N*-Acetylglucosylamine	3.36 × 10^−5^	
C_0029	2-Amino-2-(hydroxymethyl)-1,3-propanediol	1.46 × 10^−4^		A_0057	*N*-Acetylmuramic acid	1.47 × 10^−4^	
C_0021	3-Amino-2-piperidone	8.60 × 10^−5^		C_0062	*N*-Methyltyramine *N*-Methylphenylethanolamine	1.20 × 10^−4^	
C_0011	3-Aminopropane-1,2-diol	1.90 × 10^−4^		C_0079	*N*^6^,*N*^6^,*N*^6^-Trimethyllysine	1.29 × 10^−5^	
C_0037	3-Guanidinopropionic acid	2.23 × 10^−5^		C_0065	*N*^6^-Methyllysine	3.68 × 10^−5^	
C_0063	4-(β-Acetylaminoethyl)imidazole	2.67 × 10^−5^		C_0030	Nicotinic acid	6.10 × 10^−5^	
C_0053	4-Guanidinobutyric acid	3.84 × 10^−5^		C_0072	Noradrenaline 6-Hydroxydopamine	4.58 × 10^−5^	
C_0006	4-Methylpyrazole	2.66 × 10^−5^		A_0067	*p*-Anisic acid *o*-Hydroxyphenylacetic acid Mandelic acid Phenoxyacetic acid	3.48 × 10^−5^	
A_0050	4-Pyridoxic acid	1.20 × 10^−3^		A_0068	*p*-Toluic acid *m*-Toluic acid *o*-Toluic acid	1.46 × 10^−5^	
C_0031	5-Methylcytosine	1.98 × 10^−5^		A_0058	Pantothenic acid	1.30 × 10^−5^	
C_0041	6-Aminohexanoic acid	2.31 × 10^−4^		C_0097	Penciclovir	2.90 × 10^−5^	
A_0051	6-Hydroxynicotinic acid	3.43 × 10^−5^		A_0059	Perillic acid	1.43 × 10^−4^	
C_0098	Adenosine	1.53 × 10^−5^	0.17	A_0060	Phthalic acid	1.00 × 10^−4^	
C_0083	ADMA	2.33 × 10^−5^		A_0061	Pimelic acid	1.95 × 10^−5^	
C_0047	Anthranilic acid	5.11 × 10^−5^	0.56	A_0062	Prostaglandin E_2_	3.96 × 10^−5^	
C_0103	Argininosuccinic acid	1.94 × 10^−5^		C_0067	Pterin	3.27 × 10^−5^	
A_0052	Azelaic acid	4.14 × 10^−5^		C_0070	Pyridoxal	3.13 × 10^−5^	
C_0081	Carbendazim	4.61 × 10^−4^		C_0071	Pyridoxamine	7.46 × 10^−5^	
C_0066	Carnitine	1.39 × 10^−3^		C_0073	Pyridoxine	2.14 × 10^−4^	
C_0080	Castanospermine	2.10 × 10^−4^		C_0107	Riboflavin	4.89 × 10^−5^	
C_0035	*cis*-4-Hydroxyproline	2.54 × 10^−5^		A_0073	*S*-Sulfocysteine	7.82 × 10^−6^	
A_0008	Citric acid	4.36 × 10^−5^	1.60	C_0101	Saccharopine	1.25 × 10^−4^	
C_0094	Cytidine	2.00 × 10^−5^	0.30	C_0084	SDMA	2.00 × 10^−5^	
C_0018	Cytosine	4.98 × 10^−4^	11.22	C_0092	Ser-Glu	1.30 × 10^−5^	
A_0053	Decanoic acid	2.02 × 10^−5^		A_0063	Suberic acid	2.88 × 10^−5^	
C_0017	Diethanolamine	4.49 × 10^−4^		C_0069	Taurocyamine	4.62 × 10^−4^	
C_0002	Ethanolamine	3.02 × 10^−4^		A_0064	Terephthalic acid	7.68 × 10^−5^	
A_0069	Ethyl glucuronide	3.33 × 10^−5^		C_0026	Thr	7.14 × 10^−5^	1.21
A_0054	Formiminoglutamic acid	6.04 × 10^−5^		C_0093	Thymidine	5.22 × 10^−4^	21.33
A_0055	Formylanthranilic acid	4.63 × 10^−5^		C_0060	Triethanolamine	3.57 × 10^−4^	
C_0077	Galactosamine Glucosamine	1.93 × 10^−4^		C_0001	Trimethylamine	1.27 × 10^−4^	
C_0106	Gibberellic acid	2.47 × 10^−4^		C_0004	Trimethylamine *N*-oxide	7.15 × 10^−5^	
C_0058	Glu	3.21 × 10^−5^	0.72	C_0095	Uridine	1.40 × 10^−4^	6.25
A_0010	Gluconic acid	1.65 × 10^−5^	1.09	C_0049	Urocanic acid	2.34 × 10^−4^	
C_0082	Glucosaminic acid	1.39 × 10^−5^		A_0065	Vanillic acid	2.85 × 10^−5^	
A_0070	Glucuronic acid-1 Galacturonic acid-1	3.16 × 10^−5^		A_0074	XA0003	1.29 × 10^−5^	
A_0071	Glucuronic acid-2 Galacturonic acid-2	3.29 × 10^−5^		C_0076	Xanthopterin	7.62 × 10^−5^	
C_0012	Glycerol	1.37 × 10^−3^		A_0066	Xanthosine	1.10 × 10^−5^	
C_0051	Histidinol	3.67 × 10^−5^		C_0052	XC0029	3.03 × 10^−5^	
C_0033	Imidazole-4-acetic acid	6.04 × 10^−5^		C_0088	XC0065	3.28 × 10^−5^	
C_0005	Isopropanolamine	8.90 × 10^−5^		C_0054	γ-Butyrobetaine	7.07 × 10^−5^	
A_0056	Isovaleric acid Valeric acid	2.08 × 10^−5^					

CE-TOFMS analysis was used as described in the Section 4. Substances detected in cation mode are indicated by an ID of C_ and those detected in anion mode are indicated by an ID of A_. Substances involved in hair growth are shown in gray color and those related to hair care are shown in diagonal lines.

**Table 2:** Top 20 variable genes changed by MXD at 1W, 2W, and 3W post-treatment.

MXD 1W				MXD 2W				MXD 3W			
Gene Name	Average Fold	Gene Name	Average Fold	Gene Name	Average Fold	Gene Name	Average Fold	Gene Name	Average Fold	Gene Name	Average Fold
*Cxcl5*	89.81	*Myh7*	0.05	*Kcnk12*	7.12	*Cyp2a5*	0.16	*Slc10a4*	4.00	*Slc22a2*	0.25
*Saa3*	29.25	*Hamp*	0.13	*Entpd8*	6.60	*Rab39*	0.19	*Dppa3*	3.30	*Ifng*	0.30
*Cxcl3*	14.29	*Myh7*	0.18	*Syt4*	5.56	*Trim34a*	0.21	*Krtap19-1*	3.06	*Dnah17*	0.32
*Sprr2f*	13.23	*Hamp2*	0.19	*Plb1*	4.88	*Ifng*	0.22	*Sele*	3.00	*Caln1*	0.33
*Chrng*	10.93	*Apol7a*	0.19	*Gltpd2*	4.87	*Cpb1*	0.22	*Krtap19-4*	2.87	*Slc12a1*	0.33
*Reg1*	10.35	*Gbp2b*	0.21	*Capn8*	4.49	*Hs1bp3*	0.23	*Dhrs2*	2.76	*Slc5a2*	0.33
*Stfa2l1*	9.73	*Myl2*	0.22	*Pax6*	4.34	*Krt2*	0.23	*Stfa2l1*	2.73	*Gzma*	0.34
*Gm5483*	8.99	*Maz*	0.28	*Slc26a3*	4.31	*Ccdc37*	0.24	*Nrg1*	2.71	*Klra7*	0.35
*Sprr2d*	8.70	*Cpb1*	0.28	*Hcn3*	4.30	*Pkib*	0.24	*Grp*	2.66	*Cyp2c44*	0.35
*Stfa2*	8.29	*Serpina3f*	0.29	*Epb4.1*	4.30	*Lgi1*	0.24	*Sv2b*	2.61	*Folr4*	0.36
*Ankrd1*	8.20	*Lix1*	0.29	*Efr3a*	4.16	*Sox2ot*	0.25	*Stfa2*	2.60	*Foxc2*	0.37
*Gdf6*	7.81	*Myl10*	0.30	*Pcdh8*	4.04	*Skint11*	0.25	*Olfr577*	2.59	*Htra4*	0.37
*Lce3f*	7.44	*Noxa1*	0.30	*Syce1*	3.89	*Fam19a3*	0.26	*Olfr692*	2.57	*Zc3h6*	0.37
*S100a8*	7.42	*Tnnt2*	0.31	*Ret*	3.84	*Serpinb10*	0.26	*Gpr126*	2.56	*Olfr644*	0.37
*Stfa1*	7.17	*Ces2b*	0.31	*Sv2a*	3.81	*Serpinb3a*	0.26	*Nudcd1*	2.55	*Il1rl1*	0.37
*Krt16*	6.89	*Reep3*	0.32	*Foxj1*	3.80	*Msln*	0.26	*Hsf2*	2.52	*Esm1*	0.38
*Prg4*	6.84	*Osbpl6*	0.33	*Lef1*	3.76	*Mucl1*	0.26	*Krtap16-1*	2.50	*Prr18*	0.39
*Reg3g*	6.81	*Dixdc1*	0.33	*Cyb5r2*	3.76	*Krt2*	0.26	*Spink6*	2.49	*Col9a3*	0.39
*Myh3*	6.76	*F2*	0.33	*Dsg4*	3.75	*Apof*	0.26	*Pcsk1*	2.49	*H2-Ob*	0.40
*Myh3*	6.74	*Ap1s3*	0.34	*Ammecr1*	3.75	*Gm4788*	0.27	*Krtap19-1*	2.47	*Ebf4*	0.40

**Table 3:** Top 20 variable genes changed by N-CE at 1W, 2W, and 3W post-treatment.

N-CE 1W				N-CE 2W				N-CE 3W			
Gene Name	Average Fold	Gene Name	Average Fold	Gene Name	Average Fold	Gene Name	Average Fold	Gene Name	Average Fold	Gene Name	Average Fold
*Ccr3*	4.87	*Myh7*	0.03	*Myh7*	4.88	*Il10*	0.30	*Lpp*	6.11	*Slc15a2*	0.20
*Phgr1*	3.86	*Syt4*	0.06	*Myh7*	4.77	*Il10*	0.33	*Gm7361*	4.59	*Ccdc37*	0.28
*Misp*	3.82	*Myh7*	0.06	*Lhx9*	3.42	*Olfr1384*	0.35	*Gpatch2*	3.36	*Hamp*	0.29
*Prg2*	3.81	*Krtap28-10*	0.09	*Fos*	3.25	*Timd2*	0.36	*H2afz*	3.15	*Gbp2b*	0.32
*Scgb1b27*	3.72	*Gja3*	0.09	*Fos*	3.24	*Olfr33*	0.37	*Sez6*	3.10	*Paxip1*	0.33
*Olfr619*	3.55	*Gm7544*	0.11	*Fos*	3.21	*Kif11*	0.37	*Rcbtb2*	3.07	*Serpinb10*	0.33
*Fam163b*	3.51	*Otop2*	0.11	*Fos*	3.21	*Gpr27*	0.38	*Pcdhb10*	2.79	*Ifng*	0.34
*Klra2*	3.43	*Syt4*	0.11	*Nek5*	3.19	*Olfr578*	0.39	*Cyp2j11*	2.70	*Rhox9*	0.35
*Fabp9*	3.29	*Krtap4-13*	0.11	*Fos*	3.18	*Kcnn2*	0.39	*F12*	2.63	*Fpr2*	0.36
*Tnfrsf14*	3.17	*Shh*	0.12	*Fos*	3.15	*Krt2*	0.40	*Mia2*	2.56	*Klrc3*	0.36
*Cftr*	3.15	*Adamts19*	0.12	*Fos*	3.15	*Vgll1*	0.40	*Adamts19*	2.49	*Igj*	0.37
*Cabs1*	3.07	*S100a7a*	0.12	*Fos*	3.14	*Zcwpw1*	0.41	*Dhrs2*	2.43	*Tfpi2*	0.37
*Dusp15*	3.03	*Uox*	0.12	*Fos*	3.14	*Asphd1*	0.41	*Tdrd12*	2.40	*Gcsam*	0.37
*Zc3h3*	2.92	*Tchh*	0.12	*Fos*	3.12	*Podnl1*	0.41	*Bpifb3*	2.39	*Il10*	0.37
*Sec16b*	2.92	*Adamts18*	0.12	*Add2*	3.02	*Ppp1r3e*	0.42	*Olfr128*	2.38	*Olfr513*	0.37
*Fgf2*	2.91	*Oca2*	0.12	*Sim2*	2.84	*Tcp11l1*	0.42	*D17H6S53E*	2.37	*Cd28*	0.38
*Angptl3*	2.90	*Soat2*	0.13	*Pax6*	2.77	*Dhx38*	0.43	*Pbx3*	2.31	*Syce1l*	0.38
*Pde4d*	2.88	*Shh*	0.13	*Pappa*	2.74	*Cyp2c40*	0.43	*Olfr131*	2.30	*Wscd2*	0.38
*Dusp15*	2.87	*Slc7a11*	0.13	*Gabrg1*	2.71	*Krt2*	0.43	*Pard3*	2.25	*Hgf*	0.38
*Cntnap2*	2.85	*Fam26d*	0.13	*Fam187a*	2.70	*Slc4a4*	0.43	*Mgat5b*	2.23	*Gzma*	0.38

**Table 4:** A comparison of the up- and down-regulated genes in the MXD and N-CE group 1 week, 2 weeks, and 3 weeks after treatment. DNA microarray was performed as described in the Section 4.

MXD/Cont (Fold Change Values (LOG2))						N-CE/Cont (Fold Change Values (LOG2))					
Symbol	1W	Symbol	2W	Symbol	3W	Symbol	1W	Symbol	2W	Symbol	3W
*S100a9*	7.03	*Krt27*	3.11	*Epg5*	16.10	*Ascc3*	2.44	*Plcb4*	3.79	*Vps51*	28.09
*Ngp*	6.16	*Krt25*	3.03	*Borcs6*	4.38	*Tcte2*	2.01	*Fam25c*	3.43	*Dnajc16*	19.23
*Krt6b*	4.21	*Krt28*	2.96	*Nf1*	3.37	*Col11a1*	1.95	*Ect2*	3.09	*Prkar1a*	5.65
*Klf10*	3.00	*Krt26*	2.85	*Sptlc2*	3.36	*Gm45927*	1.75	*Epg5*	3.07	*Sil1*	5.19
*Abcb9*	2.86	*Pinlyp*	2.78	*Krt25*	2.84	*Ldb1*	1.74	*Arr3*	2.73	*Smarcc1*	5.11
*Fgg*	2.52	*Tchh*	2.76	*Krt27*	2.71	*Fam117b*	1.71	*Timm13*	2.70	*Cdc42bpg*	3.95
*Fgb*	2.42	*Nf1*	2.64	*Naa40*	2.59	*Xpo4*	1.71	*Smc2*	2.70	*Sptlc2*	3.73
*Fga*	2.19	*Crym*	2.58	*Fbp1*	2.57	*Gm10964*	1.66	*Lig3*	2.64	*Ston1*	3.67
*Vps51*	2.17	*Fbp1*	2.29	*Tchh*	2.47	*Hrnr*	1.64	*Uqcc2*	2.43	*Borcs6*	3.29
*Pla2g4b*	2.14	*Cgnl1*	2.19	*Pinlyp*	2.43	*Vps35l*	1.63	*Fuom*	2.21	*Smc2*	2.96
*Dnajc16*	2.09	*Tchhl1*	2.18	*Crym*	2.36	*Alox8*	1.63	*Cgn*	2.00	*Manba*	2.75
*Myo1e*	1.96	*Padi3*	2.16	*Tchhl1*	2.29	*Krt6b*	1.62	*S100a6*	1.90	*Lig3*	2.69
*Commd3*	1.78	*Padi1*	2.12	*Krt28*	2.26	*Kctd1*	1.58	*Elmo2*	1.90	*Myo5a*	2.66
*Tmem50a*	1.66	*Padi4*	2.10	*Krt26*	2.25	*Pla2g4b*	1.57	*Commd3*	1.88	*Epb41l2*	2.61
*Prkar1a*	1.63	*Prr9*	2.09	*Padi4*	2.22	*Itpkc*	1.55	*Cgnl1*	1.85	*Borcs5*	2.53
*Pafah1b2*	1.61	*Map3k10*	2.08	*Map3k10*	2.17	*Rad50*	1.54	*Selenom*	1.85	*Degs1*	2.51
*Ascc3*	1.59	*Crnn*	2.08	*Cbs*	2.11	*H2ac20*	1.50	*Slc12a4*	1.83	*Mfap5*	2.45
*Fn1*	1.54	*Krtap15*	2.06	*Krtap15*	2.10	*Fxyd1*	1.42	*Agrn*	1.82	*Cstf3*	2.40
*Tcte2*	1.54	*Cbs*	2.04	*Gnmt*	2.09	*Itgb3*	1.41	*Ahcyl2*	1.77	*Ascc3*	2.38
*Lyz2*	1.53	*Mab21l4*	2.01	*Rpl24*	2.07	*Borcs6*	1.39	*Banf1*	1.75	*Acp5*	2.37
*Plcb4*	−2.33	*Tnnc2*	−1.76	*Vps51*	−5.64	*Arr3*	−9.32	*Gm10964*	−2.15	*Tmem87a*	−2.46
*Myl1*	−1.91	*Myl1*	−1.71	*Dnajc16*	−2.80	*Smc2*	−7.52	*Krtap21-1*	−1.52	*Cyfip2*	−2.25
*Arr3*	−1.82	*Col11a1*	−1.70	*4930562C15Rik*	−2.08	*Ect2*	−4.86	*Lgals3bp*	−1.50	*Scyl1*	−2.16
*Tnnc2*	−1.72	*Vps51*	−1.60	*Ston1*	−2.04	*Acp5*	−3.33	*Krt76*	−1.50	*Ephx3*	−2.10
*Atp2a1*	−1.65	*4930562C15Rik*	−1.60	*Mybpc1*	−1.97	*Cgn*	−3.24	*Gm11567*	−1.49	*Gm45927*	−2.09
*Ccdc127*	−1.65	*Kctd1*	−1.56	*Rad50*	−1.91	*Plcb4*	−3.14	*Gm45927*	−1.44	*S100a11*	−2.06
*Trdn*	−1.63	*Krt76*	−1.56	*Pacsin2*	−1.87	*Slc12a4*	−3.10	*Gm11938*	−1.43	*Sepsecs*	−2.04
*Spag7*	−1.57	*Nucks1*	−1.46	*Ttn*	−1.73	*Lig3*	−2.97	*Pla2g4b*	−1.43	*S100a6*	−1.99
*Calm3*	−1.55	*Tsc22d1*	−1.44	*Smc5*	−1.73	*Nf1*	−2.70	*Gm11565*	−1.40	*Car13*	−1.98
*4930562C15Rik*	−1.54	*Tgfbi*	−1.43	*Tnni2*	−1.66	*Fam25c*	−2.35	*Rgs13*	−1.40	*Epb41*	−1.97
*Epg5*	−1.53	*Ncdn*	−1.43	*Serpinb7*	−1.66	*Rfc4*	−2.19	*Dera*	−1.40	*Gng12*	−1.94
*Rgs13*	−1.52	*Rad50*	−1.43	*Plcl1*	−1.64	*Agrn*	−2.08	*Tcte2*	−1.39	*Get4*	−1.93
*Pvalb*	−1.52	*Gm45927*	−1.42	*Atp2a1*	−1.63	*Vps33b*	−2.07	*Rad50*	−1.38	*Ccdc127*	−1.93
*Selenom*	−1.50	*Slc25a4*	−1.41	*Atp2a3*	−1.60	*Manba*	−1.99	*Cnbp*	−1.36	*Tsc22d1*	−1.92
*Rpl24*	−1.49	*Mcpt4*	−1.40	*Eno1*	−1.59	*Krt76*	−1.93	*Col11a1*	−1.35	*Cav3*	−1.89
*Scd1*	−1.48	*Apobec2*	−1.40	*Lmod3*	−1.58	*Armt1*	−1.92	*Eno2*	−1.34	*Plcl1*	−1.88
*Plcl1*	−1.48	*Trim16*	−1.40	*Slc25a4*	−1.58	*Borcs5*	−1.89	*Sdhc*	−1.32	*Eno2*	−1.84
*Sdhc*	−1.47	*Mapt*	−1.40	*Uggt1*	−1.58	*Epg5*	−1.87	*Sos1*	−1.31	*Itpkc*	−1.83
*Fabp3*	−1.46	*Calm3*	−1.38	*Ndufa3*	−1.57	*Hmcn1*	−1.85	*Kctd1*	−1.30	*Hook1*	−1.80
Atp2a3	−1.45	Tnni2	−1.38	*Myh1*	−1.57	*Fam83h*	−1.84	Mb	−1.30	Commd9	−1.79

**Table 5:** A biofunctional categorization and pathway of the identified genes and proteins in the MXD group 1 week, 2 weeks, and 3 weeks after treatment. Both the up- and down-regulated terms for genes and proteins were displayed together for comparison. DNA microarray and proteomics and DAVID analyses were performed as described in the Section 4.

MXD—UP: Microarray			MXD—UP: Proteome		
1W	2W	3W	1W	2W	3W
Keratin	Keratin	Keratin	Keratin	Keratin	Keratin
Disulfide bond	Cell division	Melanin biosynthesis	Intermediate filament	Intermediate filament	Intermediate filament
Keratinization	Cell cycle	Albinism	Lysosome	Lipid biosynthesis	Fatty acid metabolism
Collagen	Intermediate filament	Kinetochore	Lipid metabolism	Lipid metabolism	Protein phosphatase
Inflammatory response	Citrullination		Acyltransferase	Fatty acid metabolism	Lysosome
Chemotaxis	Microtubule		Cell adhesion	Cell cycle	Fatty acid biosynthesis
Cell adhesion	Centromere		Hemostasis	Acyltransferase	Lipid metabolism
Myosin	Melanin biosynthesis		Blood coagulation	Lysosome	Pyridoxal phosphate
Intermediate filament	Disease mutation		Cysteine biosynthesis	Fatty acid biosynthesis	Lipid biosynthesis
Cytokine	Wnt signaling pathway		Cytoskeleton	Pyridoxal phosphate	Cysteine biosynthesis
Melanin biosynthesis	Keratinization		Fatty acid metabolism	Protein phosphatase	Cell junction
Acute phase	DNA repair		Disulfide bond	Cell junction	
Albinism	Actin-binding		Amino-acid biosynthesis	Cysteine biosynthesis	
Amidation	Homeobox			Cytoskeleton	
Proteoglycan	Albinism			Heparin-binding	
Antimicrobial	Lipid-binding			Cell division	
	Zinc transport			Endoplasmic reticulum	
	Tyrosine-protein kinase			Proteoglycan	
**MXD—Down: Microarray**			**MXD—Down: Proteome**		
**1W**	**2W**	**3W**	**1W**	**2W**	**3W**
Muscle protein	Disulfide bond	Disulfide bond	Muscle protein	Muscle protein	Muscle protein
Lipid metabolism	Muscle protein	Muscle protein	Keratin	Endocytosis	Calmodulin-binding
Lipid biosynthesis	Sarcoplasmic reticulum	Actin-binding	Intermediate filament	Sarcoplasmic reticulum	Myosin
Thick filament	Lectin	Sarcoplasmic reticulum	Myosin	Thick filament	Respiratory chain
Fatty acid metabolism	Actin-binding	Cell adhesion	Thick filament	Calmodulin-binding	Sarcoplasmic reticulum
Calmodulin-binding	Cytokine	Collagen	Calmodulin-binding	Myosin	Thick filament
Oxidoreductase	Inflammatory response	Thick filament	Actin-binding	Lysosome	Actin-binding
Sarcoplasmic reticulum	Myogenesis	Growth factor	Metal-binding	Actin-binding	Cytoskeleton
Actin-binding	Calmodulin-binding	Sugar	Endoplasmic reticulum	Mitochondrion	Metal-binding
Myosin	Lipoprotein	Heme	Endocytosis	Cytoskeleton	Lysosome
Fatty acid biosynthesis	Thick filament	Calmodulin-binding	Cell division		Prenylation
Heme	Growth factor		Iron		Disulfide bond
Growth factor	Heme		Lysosome		
Lipid	Lipid metabolism				
Disulfide bond	Myosin				
Acylferase	Chemotaxis				
Lipoprotein	Osteogenesis				
Glucose metabolism	Heparin-binding				

**Table 6:** A biofunctional categorization and pathway of the identified genes and proteins in the N-CE group 1 week, 2 weeks, and 3 weeks after treatment. Both the up- and down-regulated terms for genes and proteins were displayed together for comparison. DNA microarray and proteomics and DAVID analyses were performed as described in the Section 4.

N-CE—UP: Microarray			N-CE—UP: Proteome		
1W	2W	3W	1W	2W	3W
Immunity	Homeobox	Citrullination	Keratin	Keratin	Keratin
Lectin	Disease mutation	Cell adhesion	Intermediate filament	Lysosome	Intermediate filament
Disulfide bond	Developmental protein	Disease mutation	Metal-binding	Lipid biosynthesis	Lysosome
Innate immunity	Spermatogenesis	Acetylation	Calmodulin-binding	Cytoskeleton	Cytoskeleton
Cytokine	Zymogen	Homeobox	Hemostasis	Cell adhesion	Keratinization
Inflammatory response			Blood coagulation	Lipid metabolism	Calmodulin-binding
Chemotaxis			Muscle protein	Fatty acid metabolism	Cysteine biosynthesis
Antiviral defense			Actin-binding	Cell cycle	Proteoglycan
Acute phase			Cytoskeleton	Sarcoplasmic reticulum	Myosin
Golgi apparatus			Disease mutation	Cysteine biosynthesis	Actin-binding
			Myosin	Intermediate filament	Acyltransferase
**N-CE—Down: Microarray**			Endocytosis	Proteoglycan	Aminopeptidase
**1W**	**2W**	**3W**	Lipid-binding	Myosin	Protein phosphatase
Keratin	Lectin	G-protein coupled		Prenylation	
Cell cycle	Disulfide bond	Disulfide bond		Cell division	
Mitosis	Muscle protein	Olfaction			
Cell division	Pheromone-binding	Sensory transduction	**N-CE—Down: Proteome**		
Kinetochore	Calmodulin-binding	Cell adhesion	**1W**	**2W**	**3W**
Intermediate filament	Oxygen	Sulfation	Keratin	Keratin	Muscle protein
Microtubule	Lipoprotein	Lectin	Fatty acid metabolism	Intermediate filament	Metal-binding
DNA repair		Adaptive immunity	Lysosome	Muscle protein	Sarcoplasmic reticulum
DNA replication		Heme	Intermediate filament	Lipid-binding	Fatty acid metabolism
Melanin biosynthesis		Hormone	Lipid biosynthesis	Metal-binding	Fatty acid biosynthesis
Calmodulin-binding		Immunity	Muscle protein	Myosin	Lipid biosynthesis
Citrullination		Iron transport	Fatty acid biosynthesis	Endocytosis	Lipid metabolism
Albinism		Homeobox	Lipid metabolism	Keratinization	Endocytosis
Wnt ing pathway		Myogenesis	Cysteine biosynthesis	Actin-binding	Keratin
Helicase		Cytokine	Cell cycle	Calmodulin-binding	
Osteogenesis		Zymogen	Cell adhesion		
Muscle protein		Actin-binding	Proteoglycan		

## Data Availability

The data presented in this study are available in the article and submitted databases. The raw data are available upon a reasonable request from the corresponding author.

## Supplementary Materials

The following supporting information can be downloaded at: https://www.mdpi.com/article/10.3390/molecules28041565/s1, Supplementary Table S1. Identification of differentially modulated proteins by TMT-based quantitative proteomic analysis.

## Sample Availability

Samples of the compounds are not available from the authors.
